# Improving children's nutrition environments: A survey of adoption and implementation of nutrition guidelines in recreational facilities

**DOI:** 10.1186/1471-2458-11-423

**Published:** 2011-06-01

**Authors:** Dana Lee Olstad, Shauna M Downs, Kim D Raine, Tanya R Berry, Linda J McCargar

**Affiliations:** 1 Alberta Institute for Human Nutrition, 2-021D Li Ka Shing Centre, University of Alberta, Edmonton, AB T6G 2E1, USA; 2 Department of Agricultural, Food and Nutritional Science, 4-10 Agriculture/Forestry Centre, University of Alberta, Edmonton, AB T6G 2P5, USA; 3 Centre for Health Promotion Studies, 1001 College Plaza, Edmonton, AB T6G 2C8, USA; 4 Faculty of Physical Education and Recreation, 6-37 General Services Building, University of Alberta, Edmonton, AB T6G 2H1, USA

**Keywords:** childhood obesity, childhood obesity prevention, recreational facilities, food environment, nutrition guidelines, Alberta Nutrition Guidelines for Children and Youth

## Abstract

**Background:**

Although the mandate of recreational facilities is to enhance well-being, many offer foods inconsistent with recommendations for healthy eating. Little is known regarding recreational facility food environments and how they might be improved, as few studies exist. The Alberta Nutrition Guidelines for Children and Youth (ANGCY) are intended to ensure access to healthy food choices in schools, childcare and recreational facilities. This study investigated awareness, adoption and implementation of the ANGCY among recreational facilities in Alberta, Canada, one year following their release.

**Methods:**

A cross-sectional telephone survey was conducted from June - December, 2009 (n = 151) with managers of publicly funded recreational facilities that served food. The questionnaire included 10 closed and 7 open ended questions to assess the organizational priority for healthy eating, awareness, adoption and implementation of the ANGCY. Chi-squared tests examined quantitative variables, while qualitative data were analysed using directed content analysis. Greenhalgh's model of diffusion of complex innovations within health service organizations constituted the theoretical framework for the study.

**Results:**

One half of respondents had heard of the ANGCY, however their knowledge of them was limited. Although 51% of facilities had made changes to improve the nutritional quality of foods offered in the past year, only a small fraction (11%) of these changes were motivated by the ANGCY. At the time of the survey, 14% of facilities had adopted the ANGCY and 6% had implemented them. Barriers to adoption and implementation were primarily related to perceived negative attributes of the ANGCY, the inner (organizational) context, and negative feedback received during the implementation process. Managers strongly perceived that implementing nutrition guidelines would limit their profit-making ability.

**Conclusions:**

If fully adopted and implemented, the ANGCY have the potential to make a significant and sustained contribution to improving the recreational facility food environment, however one year following their release, awareness, adoption and implementation of the ANGCY remained low. A mandated policy approach could offer an efficacious, cost-effective means of improving the food environment within recreational facilities.

## Background

It is widely recognized that the childhood obesity epidemic is primarily driven by unhealthy environments that promote consumption of energy-rich, nutrient-poor foods, and that discourage physical activity [1]. The food or nutrition environment refers to the context in which consumers purchase food, including the availability, cost, quality and promotion of healthy and unhealthy food choices [2]. The current food environment has been described as unhealthy and even obesogenic [3] because energy-dense and nutrient-poor foods are readily available, inexpensive, convenient, and heavily promoted. A predominance of unhealthy food environments has contributed to a high prevalence of dietary behaviours that increase the risk of obesity among children [3]. School-based studies, by contrast, have shown that healthy food environments foster good dietary behaviours [4-7] and appropriate body weights among children [5,6] and as such, there is significant momentum across North America to improve school nutrition environments. Many, however, overestimate time spent in school, which in reality accounts for 20% of children's waking hours over the course of a year [8]. That this figure leaves 80% of time unaccounted for suggests a need to focus on obesity prevention in other settings [8]. The current focus on school-based initiatives ignores the broader context of unhealthy food environments [9] where less advantaged children, in particular, continue to be exposed to conditions that promote unhealthy dietary behaviours [10,11]. A predominance of healthy food environments throughout communities will help to reinforce healthy eating behaviours (i.e. eating patterns consistent with recommendations in *Eating Well with Canada's Food Guide*) learned in school, ensure that intake of less healthful foods (i.e. foods with a high calorie, fat, sugar and/or sodium content, and a low micronutrient content) is not displaced from school to community food environments, and maintain and accrue further health benefits.

Although the mandate of recreational facilities is to enhance well-being, many offer foods inconsistent with recommendations for healthy eating [12-14]. To this end, municipalities are being encouraged to improve the nutrition environment within recreational facilities [15-20], and some mandated policies now exist [21,22]. The Canadian province of Alberta has included voluntary nutrition guidelines for recreational facilities in the Alberta Nutrition Guidelines for Children and Youth (ANGCY). Released in June, 2008, the ANGCY are intended to promote child health in Alberta by equipping facilities and organizations with the tools they need to ensure children and youth have access to healthy food choices within a variety of settings, including schools, childcare and recreational facilities [17].

There are very few published studies of recreational facility food environments [12-14,23-25]. Additional data would be timely and relevant, as several jurisdictions have recently initiated action to improve recreational facility food environments [17,21-24,26,27]. The ANGCY represent a novel public health intervention with relevance for health policy in many nations. Therefore, we sought to investigate whether, and to what extent recreational facilities in Alberta were aware of, and had adopted and implemented the ANGCY, and the barriers to their adoption and implementation. We define awareness as having knowledge of the ANGCY, adoption as a one-time mental decision to follow the ANGCY, whereas implementation refers to multiple acts that must be repeated over time to put the decision into practice [28]. The specific objectives of this study were three-fold: 1) to describe the organizational priority for healthy eating, 2) to assess awareness of, adoption and implementation of the ANGCY and 3) to describe barriers to adopting and implementing the ANGCY in recreational facilities.

## Methods

### Theoretical framework

Greenhalgh's multi-tiered model of diffusion of complex innovations within health service organizations (Table 1) and Prochaska and Velicer's transtheoretical model of change [29] constituted the theoretical framework for the study, and were used as a basis to structure data generating, analysis and interpretation [30]. Developed on the basis of an extensive meta-narrative review drawing on literature from 13 research traditions, Greenhalgh's framework identifies nine key domains in which factors influencing diffusion are found. These domains encompass many aspects of Rogers' Diffusion of Innovations theory, while excluding those that have little empirical support [30]. Innovations in public health are increasingly comprised of complex, multicomponent interventions and policies, where the unit of adoption is a group or organization [31], and therefore adoption and implementation of nutrition guidelines within recreational facilities was viewed as an appropriate context in which to apply Greenhalgh's model.

**Table 1:** Major components of the theoretical framework [30]

	Framework components	Description
	Attributes of the innovation	Perceived attributes of the innovation explain much of the variance in adoption rates.

Elements of the user system	Organizational antecedents for innovation	General features of the organization that make it more or less innovative.

	Organizational readiness for innovation	Factors that influence the organization's readiness and/or willingness to adopt a specific innovation.

	Adopter characteristics	Characteristics of adopters and their interactions with the innovation in the adoption process.

	Implementation process	Specific steps taken to put the adoption decision into practice.

	Processes of assimilation	Assimilation is a lengthy process, encompassing adoption and implementation. It is not linear, organizations may move back and forth between initiation, development and implementation of the innovation.

	Communication and influence Diffusion and dissemination	The means of spreading the innovation lie on a continuum from passive diffusion to active dissemination.

	Outer context	External influences on the organization's decision to adopt an innovation and efforts to implement it.

	Linkage among components of the model	Connections that facilitate movement of the innovation from the resource system to the user system.

Stage of change is the central organizing construct of the transtheoretical model of change, which describes behaviour change as a progression through a series of five stages [29,32]. During the first two stages of pre-contemplation and contemplation there is a movement from not intending to take action to change to considering it. In the preparation stage, action is intended in the very near future or small changes may have already been made. Those in the action stage have made change less than 6 months ago, while those in the maintenance stage have made change more than 6 months ago and are working to sustain it. Although originally developed to describe the behaviour of individuals, the model has been applied to the field of organizational change on the basis that change in individual organizational member's behaviour is the core of organizational change [32]. The theory can be readily integrated with diffusion theory as early adopters are more likely to be in later stages of change. The theory was therefore used within the current study to determine whether, and to what extent recreational facilities had adopted and implemented the ANGCY.

### Data collection

A cross-sectional telephone survey was conducted from June-December, 2009 with managers of publicly funded recreational facilities in the province of Alberta, Canada. This timing was important to capture an early perspective of adoption and implementation of the ANGCY (approximately one year following their release). Letters were sent to a random selection of 408 of the approximately 1275 publicly funded recreational facilities in Alberta informing them about the study and inviting their participation. Facilities were eligible if they provided food through vending machines and/or concession-based food service. Interviewers called each facility and asked to speak with the facility director or manager, as they were likely to have made decisions pertaining to adoption and implementation of the ANGCY and would be better able to respond from an organizational perspective. When facility managers were unavailable, another manager who could provide this perspective was interviewed. A maximum of three attempts were made to contact a representative from each facility within the study time frame. The same three individuals conducted all of the telephone interviews. Interviewers were trained together and adhered to a standardized, structured interview protocol. Following completion of five interviews, interviewers met as a group to review the survey protocol and to discuss issues of concern.

The questionnaire was designed to be completed in less than 10 minutes and included 10 closed and 7 open ended questions to assess the organizational priority for healthy eating, awareness of, adoption, and implementation of the ANGCY using a diffusion lens [28,30]. The first section was oriented around the hypothesis that facilities that placed a greater priority on healthy eating, as indicated by the presence of nutrition policies and initiatives to improve the nutrition environment, would be more likely to adopt the ANGCY (diffusion domain: attributes of the innovation). The next question was a filtering question and asked whether the respondent had heard of the ANGCY. The survey was stopped at this point if they had not. Subsequent questions focussed on the involvement of a champion and how participants first learned of the ANGCY (diffusion domain: communication and influence). Diffusion theory describes adoption and implementation as staged processes, and therefore we sought to characterize the stage of organizational change by asking whether respondents had made ANGCY-motivated changes, and to describe their intent-to-use the ANGCY in terms of the stages of change construct [29]. Facilities in stages 3-5 (preparation, action and maintenance) were classified as adopters, while those in stages 3-5 who had made ANGCY motivated change were deemed to have implemented the ANGCY. Most of these closed ended questions requested respondents to choose among three options: yes, no, or unsure, and were phrased using language from the Alberta Heart Health Organizational Capacity Survey [33]. Open ended questions were intended to elicit participant's free-flowing ideas regarding factors that influenced their adoption and/or implementation of the ANGCY, and to obtain additional details regarding their responses to closed ended questions, and were therefore not theory-based. In some cases, responses to filter questions determined whether subsequent contingency questions would be asked [34], thereby providing a purposeful sample for several questions.

Content validity (appropriateness of constructs, language, length, clarity, organization) was established based on expert review by two scientists who were involved in the development of the ANGCY and by four experts in public health nutrition, childhood obesity, psychometrics and the recreational facility environment. Upon their recommendation, small changes were made to rephrase some questions and to the order in which questions were asked. A copy of the complete survey can be accessed in the additional file (see additional file 1: Telephone Survey). The survey was reviewed and approved for use by the Human Research Ethics Board at the University of Alberta and respondents provided verbal consent to participate.

### Data analysis

Descriptive analyses were conducted to summarize all quantitative variables. Chi-squared tests examined the relationship between independent (whether someone was in charge of food service, rural/urban location, the priority of healthy eating, change in the priority of healthy eating in the past year, existence of nutrition policies, and the presence of a champion of the ANGCY) and dependent variables (awareness, adoption, and implementation). Responses of unsure were classified as missing. Analyses were performed using Stata (version 11; StataCorp LP, College Station, Texas). Results were considered significant at p < 0.05.

Greenhalgh's model provided the basis for development of a coding and categorizing scheme, and operational definitions for the codes and categories. The final scheme was inspected by an expert in health promotion and nutrition for congruence with the elements of the theoretical framework. A single investigator (DLO) used principles of directed content analysis [35]to analyse responses to open ended questions according to the theoretically-derived coding and categorizing scheme. Categories were not further integrated into themes, as this level of abstraction was not consistent with the goals of the study.

## Results

### Participation

Of the 408 facilities contacted for participation, 44 were deemed ineligible because they did not serve food or beverages. Of the remaining 364 facilities, 18 declined to participate for the following reasons; five declined because there was no one knowledgeable about food service available to answer the survey, four declined because they were not interested in completing a survey, three declined because they were undergoing renovations, and six did not provide a reason for not wanting to participate. In total, 151 facilities were reached by phone, met the inclusion criteria, and agreed to participate, representing a response rate of 41%. Twelve percent of the approximately 1275 publicly funded recreational facilities in Alberta participated in the telephone survey, however provincial officials and the Alberta Parks and Recreation Association estimate that only approximately 1020 recreational facilities in the province serve food (i.e. 80% of all publicly funded recreational facilities), therefore the true participation rate may have been closer to 15%.

### Characteristics of the study sample

**Figure 1:**
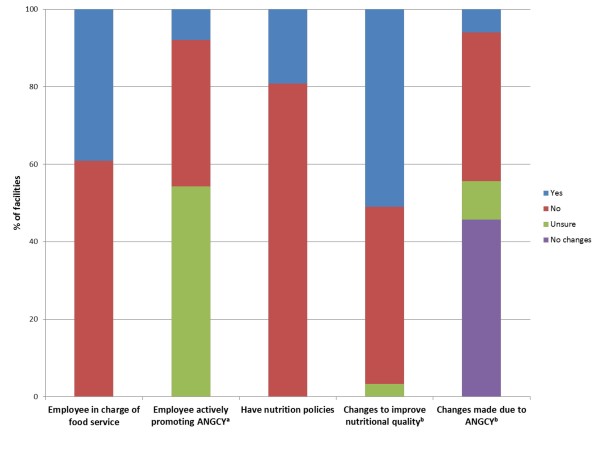
**Nutrition policies and practices of a sample of recreational facilities in Alberta (n = 151)**. ^a^This question was only asked of those who had heard of the ANGCY (n = 76), and therefore the 75 facilities that had not heard of the ANGCY were classified as "unsure". The question was worded as: "Is there someone within your organization who is very involved in promoting the guidelines?" This individual was deemed an ANGCY "champion", and may or may not have been promoting the ANGCY as part of their job-related duties. ^b^Refers to changes in the past year only. ANGCY, Alberta Nutrition Guidelines for Children and Youth.

Seventy five percent of surveys were completed by individuals employed at the managerial level within each recreational facility, whereas 18% were completed by individuals working at a managerial level or higher (eg. councillor or mayor) within the community who were knowledgeable about the food service within their local recreational facility. Notably, 39% of facilities had an individual who was responsible for food service within the organization (Figure 1). Relatively balanced representation of rural (39%) and urban (61%) facilities was achieved. To simplify reporting of results, participants are referred to as respondents or managers, although a small proportion (7%) were not managers.

### Knowledge of the ANGCY

**Figure 2:**
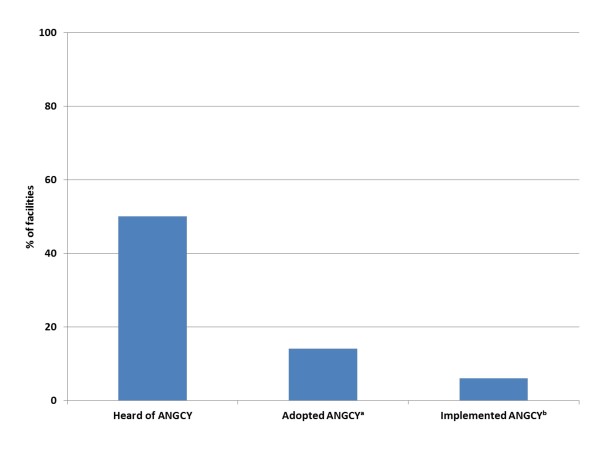
**Proportion of recreational facilities that were aware of, and had adopted and implemented the ANGCY (n = 151)**. ^a^Adoption is defined as facilities in stages 3-5 of the transtheoretical model (preparation, action, maintenance) [29]. ^b^Implementation is defined as facilities in stages 3-5 of the transtheoretical model that had made ANGCY-motivated changes to improve nutritional quality. ANGCY, Alberta Nutrition Guidelines for Children and Youth.

One half of managers in the study sample had heard of the ANGCY (Figure 2). None of the quantitative independent variables were associated with awareness of the ANGCY. The factors that contributed to knowledge of the ANGCY were found within the communication and influence domain of Greenhalgh's framework [30]. Processes of diffusion (in which spread of the ANGCY is unplanned and informal) were mediated by word of mouth (via children and adults), media and independent information seeking. Active dissemination (in which the ANGCY are spread via formal, planned strategies) occurred through formal educational events, receiving the ANGCY in the mail, emails, information provided in the workplace and via the provincial health board. The most common way that respondents who answered this question and were aware of the ANGCY (n = 66) found out about them was through the recreational facility in which they worked (n = 18), receiving the ANGCY binder in the mail (n = 11), and through word of mouth from both children and adults (n = 9). Most managers had only a limited knowledge of the content of the ANGCY.

### Priority of, and action to support healthy eating

Healthy eating was a low priority for 32% of recreational facilities, a medium priority for 50%, and a high priority for 13% (5% unsure). For most, this priority had stayed the same (50%) or had increased (44%) over the past year. This priority translated to action for 51% of facilities where specific steps had been taken in the past year to improve the nutritional quality of foods offered (i.e. foods high in essential nutrients) (Figure 1). A small fraction of these changes (11%) were motivated by the ANGCY (Figure 1).

### Nutrition policies

Nineteen percent of managers indicated that they had nutrition policies within their recreational facilities (Figure 1). These policies are summarized in Table 2. It is not clear how many policies each facility had, as managers were asked to provide examples of policies and not complete lists.

**Table 2:** Nutrition practices and policies described by a sample of managers of recreational facilities in Alberta

**Changes to improve nutritional quality**^a^	**Areas addressed by nutrition policies**^a^
Substitution of less healthy for more healthy items (eg. granola bars replace chocolate bars, baked chips replace fried chips, milk replaces soft drinks)	Availability of healthy options^b ^(eg. minimum percentage of healthy options, removing unhealthy options, substitution of healthy for unhealthy options)

Addition of healthier items (eg. sandwiches, salads, fruit, milk added to menus)	Specific nutrients and food groups (eg. no trans fats, low sugar)

Removal of less healthy items (eg. removal of chocolate bars, chips, sugar sweetened beverages)	Aesthetics (eg. healthy foods attractively and prominently displayed)

Using healthier preparation methods (eg. baking instead of frying, healthier cooking oils)	Pricing (eg. healthy foods competitively priced)

Bringing in vendors perceived to offer healthier choices (eg. Pita Pit, Booster Juice)	Provision of information (eg. menu labelling, food rating systems)
	
	Portion size (eg. reduced portion size)
	
	Allergies and food safety (eg. no nuts, no food from home)

^a^Applicable to vending machines and/or concession-based food vendors.

^b^There was a wide range in the proportion of healthy to unhealthy items permitted.

### ANGCY adoption and implementation

**Figure 3:**
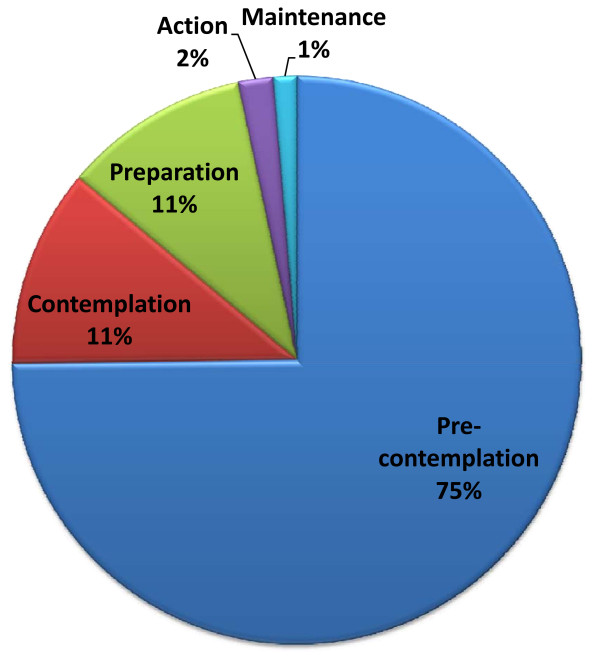
**Organizational stage of change according to the transtheoretical model (n = 151) **[29,65]. Stage 1: Pre-contemplation (have not thought about using the ANGCY); Stage 2: Contemplation (thinking about using the ANGCY); Stage 3: Preparation (planning programs and/or taking steps toward using the ANGCY); Stage 4: Action (currently promoting and using the ANGCY and have started some programs, < 6 mos); Stage 5: Maintenance (currently promoting and using the ANGCY and have started some programs, > 6 mos). ANGCY, Alberta Nutrition Guidelines for Children and Youth.

Fourteen percent of facilities were classified as adopters (Figures 2 and 3). Facilities were more likely to adopt the ANGCY if someone in their facility was actively promoting the guidelines (indicating the presence of a "champion") (p = 0.003), and if the priority for healthy eating had increased in the past year (p = 0.01) (Table 3). There was also a trend for facilities to be more likely to adopt the ANGCY if they had nutrition policies (p = 0.08).

**Table 3:** Characteristics of recreational facilities that adopted and implemented the Alberta Nutrition Guidelines for Children and Youth (n = 151)

	Proportion of facilities that adopted the ANGCY	Proportion of facilities that implemented the ANGCY
**Someone in charge of food service**	17%	9%
**Urban location**	17%	9%
**Medium to high priority of healthy eating**	16%	4%^a^
**Priority of healthy eating increased in the past year**	22%^b^	10%
**Have nutrition policies**	24%	17%^c^
**Presence of a "champion"**	67%^d^	44%^e^

^a^p < 0.05 relative to facilities that had a low priority for healthy eating (13%)

^b^p = 0.01 relative to facilities where the priority for healthy eating had not increased in the past year (8%)

^c^p < 0.05 relative to facilities that did not have nutrition policies (4%)

^d^P < 0.01 relative to facilities that did not have a "champion" (23%)

^e^p = 0.01 relative to facilities that did not have a "champion" (11%)

Six percent of facilities were deemed to have implemented the ANGCY (Figures 2 and 3). Facilities were more likely to have implemented the ANGCY if they had nutrition policies (p = 0.03) and if someone in their facility was actively promoting the guidelines (indicating the presence of a "champion") (p = 0.04) (Table 3). Conversely, facilities were less likely to implement the ANGCY if the priority for healthy eating was medium to high (p = 0.04).

### Barriers to adopting the ANGCY

Barriers to adopting the ANGCY corresponded with domains of Greenhalgh's framework related to the attributes of the innovation and the inner (organizational) context (Table 1) [30]. It is likely that barriers also existed within other domains of the framework, however the format of the interview (i.e. brief telephone survey) was more suited to uncovering more immediately evident micro and meso level barriers encountered on a daily basis, than more distal macro level barriers that may have existed within other domains of the framework.

#### 1) Attributes of the ANGCY

##### Relative advantage and risk

Managers felt very strongly that adopting the ANGCY would put them at an economic disadvantage and decrease profit. Respondents framed their responses in two distinct ways. One group spoke of the higher costs associated with supplying healthy foods, stating that it is "too expensive... to serve healthy food". The other group framed their economic concerns in terms of the demand side of the financial equation, and felt that "the products that are not healthy sell better".

##### Compatibility

Perceived incompatibility of the ANGCY with organizational mandates was an important barrier to adoption. Managers correctly perceived that the intent of the ANGCY was to improve children's dietary behaviours, whereas they described their own operations as "driven by revenue" and as "more concerned about pool safety [than about using the ANGCY]". Some resented the attempted imposition of a food-related mandate, believing that "parents should not be buying these [unhealthy foods]" and that "it [was] not up to [them] to be the food police". For others, food service was not even "on the radar". Their food service was contracted out and therefore they felt they "[did not] have control over food in [their] facilities", and that "[adoption of the ANGCY] has to be up to the providers". Therefore, to the extent that the ANGCY were perceived to promote goals that did not coincide with their own, managers regarded the ANGCY as incompatible with food service in recreational facilities.

Several managers expressed reluctance to implement the ANGCY because they either did not serve many children or served more than the child/youth demographic. Thus, these managers perceived the ANGCY to be incompatible with their customer mix.

##### Complexity

Managers perceived that the ANGCY would increase the complexity of their operations because they believed healthier foods required additional preparation time, were less convenient and had shorter shelf lives than their traditional product mixes. This complexity presented a barrier to ANGCY adoption.

#### 2) The inner (organizational) context: Organizational antecedents and readiness for the ANGCY

##### Technical capacity and dedicated time/resources

Resource limitations were perceived as problematic with respect to adopting the ANGCY. Several managers indicated that they "just have vending machines, so it is difficult to offer healthy choices", while others said that "time and staffing issues" were influential.

##### Absorptive capacity for new knowledge, managerial attitude toward change, tension for change

Cultural norms and expectations were highly influential with respect to the intent-to-use the ANGCY. Managers believed that "people love fries and burgers and that's what they want in a hockey rink", and this belief guided the provision of food. They also found it "really hard to get people to want to change", as staff and customers alike seemed content with the status quo. Thus, some managers did not use the ANGCY because they wanted to maintain organizational stability, and avoid the additional effort required to find, interpret and integrate new knowledge into the organization. They did not perceive having made a deliberate decision to serve unhealthy foods. Rather, cultural norms had become so entrenched that managers did not perceive that their food service practices were incompatible with wellness and were contributing to broader social ills. In short, they did not experience any tension for change.

#### 3) No barriers

Notably, some managers who had not adopted the ANGCY could not identify any barriers preventing them from doing so.

### Barriers to implementing the ANGCY

Barriers to implementation corresponded with elements of Greenhalgh's framework related to the attributes of the innovation and the implementation process (Table 1) [30]. Although common barriers to adoption and implementation existed (eg. profitability), the way in which participants discussed these barriers differed according to whether they were asked to describe barriers to adoption versus those affecting implementation.

#### 1) Attributes of the ANGCY

##### Compatibility

Perceived incompatibility of the ANGCY with customer expectations and profit-making emerged as a unifying theme that integrated findings from all aspects of the framework. As summarized by one manager: "Unhealthy foods are big sellers. Fried foods like french fries are cheap to buy. People have a perception of what foods they want. If people are watching a hockey game they want burgers and fries, not a salad. The operator needs to provide foods that people want. If everyone wanted salad you would make salad".

##### Relative advantage, complexity, task issues

To the extent that managers perceived that "healthier food [was] not as profitable", was less convenient, less available from wholesalers, spoiled more quickly and required more effort to prepare than unhealthy foods, they regarded their new ANGCY-inspired product mix as inferior to their previous one. These qualities of healthy items also made them more complex to work with, and less compatible with the performance of tasks than unhealthier options, further eroding managers' desire to provide healthier options.

##### Observability

Regardless of how effective the ANGCY prove to be, their contribution to improved health outcomes are primarily of a long-term nature, and recreational facility managers are unlikely to have the opportunity to observe these benefits. Although shorter-term benefits might be evident in the form of increased sales of healthy items, this was not yet the case. Instead, managers became discouraged because "healthy food was not selling", and children were instead purchasing unhealthy items from nearby convenience stores, thereby eroding their own sales.

#### 2) The implementation process

##### Feedback

Despite managers' best efforts to implement the ANGCY and incent purchase of healthy items through pricing strategies, children continued to purchase unhealthy items. These purchasing patterns acted as a form of negative feedback that suggested to managers their efforts were futile: "The other thing we have noticed is, since there is a discretionary income for kids nowadays, they will pay $8 for a poutine even if the healthier options are competitively priced. Unless we go to extreme prices that's what's going to happen".

#### 3) No barriers

Notably, some managers who had implemented the ANGCY could not identify any barriers to implementing them.

## Discussion

These findings demonstrate that awareness (50%), adoption (14%), and implementation (6%) of the ANGCY were low among this sample of recreational facilities approximately one year following their release. Similarly, evidence from the Treatment Improvement Protocols (TIPs) evaluation project suggests that awareness of government-developed best practice guidelines for substance abuse treatment spread slowly, as only 45% of professionals working in the substance abuse field were aware of the TIPs approximately seven years following their release [36]. Diffusion of tobacco control policies was also a lengthy process [37]. Initiatives to address recreational facility food environments are very recent [23,24], change will require support and thus it may not happen quickly [13,14]. Awareness of the ANGCY on the part of recreational facilities may actually be high relative to the short period of time that has elapsed since their release, and considering the fact that few resources were directed toward dissemination.

Although just over half of facilities had made changes to improve the nutritional quality of foods offered, only a small proportion (11%) of these changes were motivated by the ANGCY. This survey was not intended to assess the extent or fidelity of implementation of the ANGCY, however open-ended responses suggest that implementation was incomplete. Notably, adoption and implementation were more likely among facilities with an "ANGCY champion", a finding common in many other contexts [38,39]. Facilities with nutrition policies appeared to be more likely to adopt and implement the ANGCY, although it is not clear whether these policies were precipitated by, or existed prior to ANGCY adoption. These findings demonstrate that creating nutrition guidelines does not in itself constitute a sufficient stimulus for widespread change within the food environment of recreational facilities in the first year following their release. Similarly, awareness [40] and even adoption [41] of practice guidelines in other settings also did not guarantee their implementation. An important strength of the current study is its use of a mixed questionnaire which enabled further exploration of the distinct barriers to adoption and implementation of nutrition guidelines in this context.

It is unclear why facilities were less likely to implement the ANGCY if the priority for healthy eating was medium to high. Among the nine facilities deemed to have implemented the ANGCY, six indicated that healthy eating was a low priority. As was the case for adoption, it is possible that the change in priority is more relevant to implementation than the absolute priority, as the priority for healthy eating had increased among six of the nine implementers, and was unchanged in the other three. Furthermore, the survey was not designed to assess the extent of change made. Therefore, although these facilities had made ANGCY-motivated change, it is possible that these changes were minor, consistent with a low priority for healthy eating.

An extensive body of research supports the notion that the key attributes of innovations, as perceived by potential adopters, account for a significant proportion of the variability in adoption rates [30]. Although other factors were also important, perceived negative characteristics of the ANGCY were consistently described as barriers to their adoption and implementation. These perceptions were strongly driven by the constructs of relative advantage and compatibility [28,42], in which managers perceived that adopting and implementing the ANGCY would limit their profit-making ability. Given managers' limited knowledge of the ANGCY it is possible that some of these negative perceptions may be amenable to change through the provision of training and technical assistance [43] to enhance understanding and application of the ANGCY.

Food choices are primarily made on the basis of taste, cost and convenience, and to a lesser extent, health and variety [44,45]. Individuals vary in the importance they ascribe to each of these dimensions [44,45], however children are particularly vulnerable to external influences because they fail to take into account the future consequences of today's unhealthy dietary choices [46,47]. In this study, the perceived higher costs of healthy foods emerged as a particularly salient barrier that limited the marketability, and hence the availability, of healthier options. This finding was not surprising, as one of the most powerful ways to modify food purchases is to change food pricing [48-51]. Indeed, when healthier foods are substituted for less healthy foods at competitive prices in both cafeterias [52] and vending machines [53-55], children's purchases of healthier foods increases with no loss of revenue [56]. The threat of reduced profitability was also an important barrier to providing healthier food options in other studies of recreational facilities [13,14,23-25], however in spite of these fears, many recreational facilities intended to continue to offer healthier options [14,23,24]. This suggests that concerns related to profitability need not preclude adoption and implementation of the ANGCY.

Given that financial considerations figured prominently into the decision of managers not to adopt and implement the ANGCY, recreational facility managers could consider raising prices on less healthful foods to compensate for lowered prices of healthful options, and stipulate that food contractors do the same within negotiated contracts. This strategy may encourage substitution of healthy for unhealthy items while maintaining revenues [52,57]. In addition, environmental changes that increase availability and promotion of lower fat foods lead to greater purchase of these items among adolescents, with no adverse effects on school revenues [58]. Thus, pricing and environmental modifications analogous to those recommended in the ANGCY may act in a complementary manner to support purchase of healthy items by children without adversely affecting food service revenues. Success will, however, require a fundamental shift in the managerial role, from one in which managers simply respond to consumer demand, to one in which they endeavour to shape demand by actively manipulating food availability towards a healthier mix.

Findings from this study suggest that recreational facility managers may not recognize the contribution made by unhealthy community nutrition environments to childhood obesity. Instead, some managers held to a personal responsibility frame, holding parents responsible for what is a predictable response to toxic environmental conditions [59]. Strategies to improve problem recognition should therefore be enacted prior to proceeding further with ANGCY adoption and implementation [43].

The dissemination strategy adopted by the provincial government for recreational facilities included mailing ANGCY resource binders to municipalities, presentations by government staff at educational events and posting the guidelines on the internet. Reliance on mailings and presentations has proven ineffective in other dissemination studies [41,60-62], and appears to have had limited efficacy in this context as well. Conversely, comprehensive, resourced dissemination guided by theoretical constructs similar to those underlying the current study has been successful [41]. Awareness and uptake of the ANGCY might be improved in recreational facilities by adapting successful dissemination strategies used in other settings. It is also possible that the time frame used in this study may have been too short to see widespread awareness, adoption and implementation of the ANGCY.

## Conclusions

It is ironic that the very places where children go to be active may be perpetuating the problem of obesity by providing little access to healthy food options [12-14]. This study is one of few published accounts of adoption and implementation of nutrition guidelines in recreational facilities [14,23,24,63]. If fully adopted and implemented, the ANGCY have the potential to make a significant and sustained contribution to changing recreational facility food environments, however one year following their release, awareness, adoption and implementation of the ANGCY remained low. Findings from this study suggest that further raising the priority of nutrition, and motivating action to address the nutrition environment within recreational facilities under a voluntary approach will be a significant, resource-intensive challenge given manager's fears of reduced profitability. In contrast, a policy-based approach has significant potential to improve the nutrition environment within recreational facilities in a cost-effective and timely manner. Future studies are needed to investigate the efficacy of interventions to stimulate increased uptake of nutrition guidelines in this context, and to determine their impact on food service revenues. Recreational facilities serve large numbers of children and youth, and therefore implementation of nutrition guidelines in this setting can help to improve children's dietary behaviours at a population level.

## Limitations

It is not clear to what extent these observations reflect the entire population of publicly funded recreational facilities in Alberta, however a larger sample size was not attainable within the timelines used to define early adoption. Future studies should anticipate challenges related to contacting and recruiting rural facilities, which may rely on part-time, volunteer staff. Alberta has 1275 publicly funded recreational facilities, however the number of facilities that serve food is unknown. Provincial officials and the Alberta Parks and Recreation Association estimate that approximately 80% of the 1275 serve food, suggesting that we sampled 15% of the relevant population with a response rate of 41%. In addition, we used random sampling to provide protection against sampling bias [64]. We considered whether the inferences made would differ if our estimates were off by a margin of error of ± 7.5% with a 95% confidence interval (based on a population of 1275 recreational facilities). Under this scenario, 95% of the time the true proportion of facilities who have heard of the ANGCY would range from a low of 42% to a high of 58%. Similarly, the true proportion of adopters might range from 6% to a high of 22%, with implementers constituting between 0% and 14% of facilities. Thus, even under the worst case scenario, study findings of low levels of awareness, adoption and implementation of the ANGCY remain robust.

Given the limited nature of the qualitative data collected in this study, we were unable to fully assess the fit of Greenhalgh's framework in this context. The framework did provide a good fit for the data, however we noted several areas of overlap among subcategories, suggesting there may be areas in which the model can be rendered more concise for application in this setting. Findings from this study will be used to select a purposeful sample for a subsequent, in depth exploration of adoption and implementation of the ANGCY, thereby enabling a thorough exploration of the utility of Greenhalgh's model in this context.

## Competing interests

The authors declare that they have no competing interests.

## Authors' contributions

DLO analysed and interpreted the data and wrote the manuscript; SMD participated in the design of the study, participated in and supervised data collection, and participated in data interpretation; KDR participated in data interpretation and edited the manuscript; TRB participated in the design and implementation of the study; LJM designed the study, supervised data collection and analysis, participated in data interpretation and edited the manuscript. All authors read and approved the final manuscript.

## Pre-publication history

The pre-publication history for this paper can be accessed here:

http://www.biomedcentral.com/1471-2458/11/423/prepub

## Supplementary Material

Additional File 1 Telephone Survey, Word 97-2003 document, The Alberta Nutrition Guideline Outcomes Telephone-Survey Questionnaire, Questions asked in telephone survey Click here for file

## References

[B1] World Health Organization Obesity: Preventing and managing the global epidemic 2000 Geneva: World Health Organization 11234459

[B2] Glanz K Sallis JF Saelens BE Frank LD Healthy nutrition environments: concepts and measures Am J Health Promot 2005 19 5 330 333 ii 15895534 10.4278/0890-1171-19.5.330

[B3] Lake A Townshend T Obesogenic environments: exploring the built and food environments J R Soc Promot Health 2006 126 6 262 267 10.1177/1466424006070487 17152319

[B4] Jaime PC Lock K Do school based food and nutrition policies improve diet and reduce obesity? Prev Med 2009 48 1 45 53 10.1016/j.ypmed.2008.10.018 19026676

[B5] Veugelers PJ Fitzgerald AL Effectiveness of school programs in preventing childhood obesity: a multilevel comparison Am J Public Health 2005 95 3 432 435 10.2105/AJPH.2004.045898 15727972 PMC1449197

[B6] Foster GD Sherman S Borradaile KE Grundy KM Vander Veur SS Nachmani J Karpyn A Kumanyika S Shults J A policy-based school intervention to prevent overweight and obesity Pediatrics 2008 121 4 e794 802 10.1542/peds.2007-1365 18381508

[B7] Matson-Koffman DM Brownstein JN Neiner JA Greaney ML A site-specific literature review of policy and environmental interventions that promote physical activity and nutrition for cardiovascular health: what works? Am J Health Promot 2005 19 3 167 193 15693346 10.4278/0890-1171-19.3.167

[B8] Katz DL School-based interventions for health promotion and weight control: not just waiting on the world to change Annu Rev Public Health 2009 30 253 272 10.1146/annurev.publhealth.031308.100307 19705560

[B9] Alvaro C Jackson LA Kirk S McHugh TL Hughes J Chircop A Lyons RF Moving governmental policies beyond a focus on individual lifestyle: some insights from complexity and critical theories Health Promot Int 2010 10.1093/heapro/daq052 PMC3033735 20709791

[B10] Ransley JK Greenwood DC Cade JE Blenkinsop S Schagen I Teeman D Scott E White G Schagen S Does the school fruit and vegetable scheme improve children's diet? A non-randomised controlled trial J Epidemiol Community Health 2007 61 8 699 703 10.1136/jech.2006.052696 17630369 PMC2652997

[B11] Williams P Green R Millar N Frank L Hartleib R Working together to build food security in Nova Scotia: participatory food costing 2004/05 2007 Halifax, Nova Scotia: Atlantic Health Promotion Research Centre

[B12] Chaumette P Morency S Royer A Lemieux S Tremblay A Food environment in the sports, recreational and cultural facilities of Quebec City: a look at the situation Can J Public Health 2009 100 4 310 314 19722347 10.1007/BF03403953 PMC6973963

[B13] Naylor PJ Bridgewater L Purcell M Ostry A Wekken SV Publically funded recreation facilities: obesogenic environments for children and families? Int J Environ Res Public Health 2010 7 5 2208 2221 10.3390/ijerph7052208 20623020 PMC2898045

[B14] Naylor PJ Wekken SV Trill D Kirbyson A Facilitating healthier food environments in public recreation facilities: Results of a pilot project in British Columbia, Canada Journal of Park & Recreation Administration 2010 28 4 37 5821638770

[B15] Garcia J Beyers J Uetrecht C Kennedy E Mangles J Rodrigues L Truscott R Expert Steering Committee of the Project in Evidence-based Primary Prevention Healthy Eating, Physical Activity, and Healthy Weights Guideline for Public Health in Ontario 2010 Toronto: Cancer Care Ontario

[B16] White House Task Force on Childhood Obesity Solving the problem of childhood obesity within a generation 2010 Executive Office of the President of the United States 10.1089/bfm.2010.9980 20942695

[B17] Alberta Health and Wellness The Alberta Nutrition Guidelines for Children and Youth 2008 Edmonton, AB: Alberta Health and Wellness

[B18] Healthy eating and active living priorities of the APCCP

[B19] Government of Ontario Healthy eating, physical activity and healthy weights. Guidance document 2010 Toronto, ON: Standards, Programs & Community Development Branch. Ministry of Health Promotion. Government of Ontario

[B20] Khan LK Sobush K Keener D Goodman K Lowry A Kakietek J Zaro S Recommended community strategies and measurements to prevent obesity in the United States MMWR Recomm Rep 2009 58 RR-7 1 26 19629029

[B21] City of Hamilton Community Services Department (Culture & Recreation Division) Healthy nutritional environments in city recreational facilities (CS06015) 2006

[B22] Payton L Gatineau deep-sixing the deep fry at hockey arenas Ottawa Citizen 2009 Ottawa

[B23] Thesenvitz J Prange M Eat Smart! Recreation Centre Program Pilot Project Process Evaluation Report 2009 Toronto, ON: Nutrition Resource Centre, Ontario Public Health Association

[B24] Naylor PJ Wekken S Healthy Food and Beverage Sales in Recreation Facilities and Local Government Buildings. Process and Impact Evaluation Final Report 2009 Victoria, BC: University of Victoria

[B25] Thomas H Irwin J Food choices in recreation facilities: Operators' and patrons' perspectives Can J Diet Pract Res 2010 71 4 180 185 10.3148/71.4.2010.180 21144134

[B26] Healthy Eating Physical Activity Coalition of New Brunswick Healthy Foods in Recreation Facilities. It just makes sense 2009 Healthy Eating Physical Activity Coalition of New Brunswick

[B27] Kelly B Chapman K King L Hardy L Farrell L Double standards for community sports: promoting active lifestyles but unhealthy diets Health Promot J Austr 2008 19 3 226 228 19053941 10.1071/he08226

[B28] Rogers E Diffusion of Innovations 2003 5 New York: Free Press

[B29] Prochaska JO Velicer WF The transtheoretical model of health behavior change Am J Health Promot 1997 12 1 38 48 10170434 10.4278/0890-1171-12.1.38

[B30] Greenhalgh T Robert G Macfarlane F Bate P Kyriakidou O Diffusion of innovations in service organizations: systematic review and recommendations Milbank Q 2004 82 4 581 629 10.1111/j.0887-378X.2004.00325.x 15595944 PMC2690184

[B31] Oldenburg B Glanz K Glanz K, Rimer B, Viswanath K Diffusion of innovations Health behavior and health education Theory, research, and practice 2008 4 San Francisco: Jorsey-Bass 312 333

[B32] Prochaska JM Prochaska JO Levesque DA A transtheoretical approach to changing organizations Adm Policy Ment Health 2001 28 4 247 261 10.1023/A:1011155212811 11577653

[B33] Berry TR Plotnikoff RC Raine K Anderson D Naylor PJ An examination of the stages of change construct for health promotion within organizations J Health Organ Manag 2007 21 2 121 135 10.1108/14777260710736822 17713177

[B34] Singleton R Straits B Straits M Approaches to social research 1993 2 New York: Oxford University Press

[B35] Hsieh HF Shannon SE Three approaches to qualitative content analysis Qual Health Res 2005 15 9 1277 1288 10.1177/1049732305276687 16204405

[B36] Hubbard S Hayashi S Use of diffusion of innovations theory to drive a federal agency's program evaluation Evaluation and program planning 2003 26 49 56 10.1016/S0149-7189(02)00087-3

[B37] Nykiforuk C Eyles J Campbell H Smoke-free spaces over time: a policy diffusion study of bylaw development in Alberta and Ontario, Canada Health and Social Care in the Community 2008 16 1 64 74 18181816 10.1111/j.1365-2524.2007.00727.x

[B38] Vogel EM Burt SD Church J Case Study on Nutrition Labelling Policy-making in Canada Can J Diet Pract Res 2010 71 2 85 92 10.3148/71.2.2010.85 20525420

[B39] MacLellan D Taylor J Freeze C Developing school nutrition policies: enabling and barrier factors Can J Diet Pract Res 2009 70 4 166 171 10.3148/70.4.2009.166 19958571

[B40] Ferguson JH NIH consensus conferences: dissemination and impact Ann N Y Acad Sci 1993 703 180 198 discussion 198-189 10.1111/j.1749-6632.1993.tb26348.x 8192296

[B41] Hoelscher DM Kelder SH Murray N Cribb PW Conroy J Parcel GS Dissemination and adoption of the Child and Adolescent Trial for Cardiovascular Health (CATCH): a case study in Texas J Public Health Manag Pract 2001 7 2 90 100 12174404 10.1097/00124784-200107020-00012

[B42] Pankratz M Hallfors D Cho H Measuring perceptions of innovation adoption: the diffusion of a federal drug prevention policy Health Educ Res 2002 17 3 315 326 10.1093/her/17.3.315 12120847

[B43] Stith S Pruitt I Dees JE Fronce M Green N Som A Linkh D Implementing community-based prevention programming: a review of the literature J Prim Prev 2006 27 6 599 617 10.1007/s10935-006-0062-8 17051431

[B44] French SA Story M Hannan P Breitlow KK Jeffery RW Baxter JS Snyder MP Cognitive and demographic correlates of low-fat vending snack choices among adolescents and adults J Am Diet Assoc 1999 99 4 471 475 10.1016/S0002-8223(99)00117-0 10207402

[B45] Glanz K Basil M Maibach E Goldberg J Snyder D Why Americans eat what they do: taste, nutrition, cost, convenience, and weight control concerns as influences on food consumption J Am Diet Assoc 1998 98 10 1118 1126 10.1016/S0002-8223(98)00260-0 9787717

[B46] Institute of Medicine Food Marketing to Children and Youth: Threat or Opportunity? 2006 Washington, DC: National Academies Press

[B47] Finkelstein E French S Variyam JN Haines PS Pros and cons of proposed interventions to promote healthy eating Am J Prev Med 2004 27 3 Suppl 163 171 15450627 10.1016/j.amepre.2004.06.017

[B48] Faith MS Fontaine KR Baskin ML Allison DB Toward the reduction of population obesity: macrolevel environmental approaches to the problems of food, eating, and obesity Psychol Bull 2007 133 2 205 226 17338597 10.1037/0033-2909.133.2.205

[B49] Darmon N Ferguson E Briend A Do economic constraints encourage the selection of energy dense diets? Appetite 2003 41 3 315 322 10.1016/S0195-6663(03)00113-2 14637330

[B50] Darmon N Ferguson EL Briend A A cost constraint alone has adverse effects on food selection and nutrient density: an analysis of human diets by linear programming J Nutr 2002 132 12 3764 3771 12468621 10.1093/jn/132.12.3764

[B51] French SA Pricing effects on food choices J Nutr 2003 133 3 841S 843S 12612165 10.1093/jn/133.3.841S

[B52] Hannan P French SA Story M Fulkerson JA A pricing strategy to promote sales of lower fat foods in high school cafeterias: acceptability and sensitivity analysis Am J Health Promot 2002 17 1 1 6 ii 10.1093/heapro/17.1.1 12271753

[B53] French SA Jeffery RW Story M Breitlow KK Baxter JS Hannan P Snyder MP Pricing and promotion effects on low-fat vending snack purchases: the CHIPS Study Am J Public Health 2001 91 1 112 117 11189801 10.2105/ajph.91.1.112 PMC1446491

[B54] Fiske A Cullen KW Effects of promotional materials on vending sales of low-fat items in teachers' lounges J Am Diet Assoc 2004 104 1 90 93 10.1016/j.jada.2003.10.011 14702590

[B55] Messier P Cloutier G Rowe S Fuel to Xcell: Healthy vending machine program 2004

[B56] Wharton CM Long M Schwartz MB Changing nutrition standards in schools: the emerging impact on school revenue J Sch Health 2008 78 5 245 251 10.1111/j.1746-1561.2008.00296.x 18387023

[B57] Epstein LH Dearing KK Roba LG Finkelstein E The influence of taxes and subsidies on energy purchased in an experimental purchasing study Psychol Sci 2010 21 3 406 414 10.1177/0956797610361446 20424078

[B58] French SA Story M Fulkerson JA Hannan P An environmental intervention to promote lower-fat food choices in secondary schools: outcomes of the TACOS Study Am J Public Health 2004 94 9 1507 1512 10.2105/AJPH.94.9.1507 15333303 PMC1448482

[B59] Brownell K Horgen K Food fight: The inside story of the food industry, America's obesity crisis and what we can do about it 2004 New York: The McGraw-Hill Companies

[B60] Fowler G Mant D Fuller A Jones L The "Help Your Patient Stop" initiative. Evaluation of smoking prevalence and dissemination of WHO/UICC guidelines in UK general practice Lancet 1989 1 8649 1253 1255 2566792 10.1016/s0140-6736(89)92342-8

[B61] Mullins R Livingston P Borland R A strategy for involving general practitioners in smoking control Aust N Z J Public Health 1999 23 3 249 251 10.1111/j.1467-842X.1999.tb01251.x 10388167

[B62] Olson CM Devine CM Frongillo EA Jr Dissemination and use of a school-based nutrition education program for secondary school students J Sch Health 1993 63 8 343 348 10.1111/j.1746-1561.1993.tb07150.x 8289440

[B63] Atkinson J Healthy foods in recreational facilities': A follow up investigation 2010 New Brunswick Department of Wellness, Culture and Sport

[B64] Woodward M Epidemiology. Study design and data analysis 2005 2 New York: Chapman & Hall/CRC

[B65] Prochaska J Prochaska J Levesque D A transtheoretical approach to changing organizations Administration and Policy in Mental Health 2001 28 4 247 261 10.1023/A:1011155212811 11577653

