# Supplementary material for: Improving children's nutrition environments: A survey of adoption and implementation of nutrition guidelines in recreational facilities
Source: BMC Public Health. 2011 Jun 1;11:423. doi: 10.1186/1471-2458-11-423 (PMC3130673; doi:10.1186/1471-2458-11-423)
Supplement: Additional File 1 — Telephone Survey, Word 97-2003 document, The Alberta Nutrition Guideline Outcomes Telephone-Survey Questionnaire, Questions asked in telephone survey [file 1471-2458-11-423-S1.DOC]

**The Alberta Nutrition Guideline Outcomes Telephone-Survey Questionnaire**

Do you consent to participating in this study?

**** Yes ** **No

1. How many employees are there in your organization? _______
2. How many youth does your organization provide for? _______
3. Is there a person in charge of food service within your organization?

**** Yes **** No

1. Within your organization would you say healthy eating is a:

** **Low priority ** **Medium priority ** **High priority ** **Not sure

1. Compared to one year ago, would you say the priority given to healthy eating within your organizations has:

** **Decreased ** **Stayed the same ** **Increased ** **Not sure

1. a) Are there any current nutrition policies within your organization?

**** Yes ** **No

** **

** **b) **If Yes,** What nutrition policies currently exist within your organization?

________________________________________________________________________________________________________________________________________________________________________________________________

1. a) Have you made any changes to improve the nutritional quality of the foods

offered in your organization within the last year?

** **Yes ** **No ** **Not sure

b) If yes, please describe these changes

__________________________________________________________________________________________________________________________________________________________________________________________________________________

c) Have you heard of the Alberta Nutrition Guidelines for Children and Youth?

**** Yes **** No **(If answered no, the survey is completed here)**

1. If you made changes to improve the nutritional quality of the foods offered in your organization, were they due to the Nutrition Guidelines for Children and Youth?

** **Yes ** **No ** **Not sure ** **No changes

1. a) How did you hear about the Alberta Nutrition Guidelines for Children and Youth? ________________________________________________________________________________________________________________________________________________________________________________________________________________________________________________________
2. b) What do you know about the Alberta Nutrition Guidelines for Children and Youth?

________________________________________________________________________________________________________________________________________________________________________________________________________________________________________________________

1. a) Is there someone within your organization who is involved in promoting the Alberta Nutrition Guidelines for Children and Youth,?

** **Yes ** **No ** **Not sure

b) If Yes, what is this individual’s position in the organization?

** **Board of Directors

** **Management

** **Service-Provider

** **Other (please specify)____________________

** **Don't know

1. a) Which of the following best represents your facility’s intent-to-use the

Alberta Nutrition Guidelines for Children and Youth?

- i) We have not thought about it

** **ii) We are thinking about it

- iii) We are in preparation (planning programs and/or taking some steps)
- iv) We are currently promoting and using the guidelines and have started some programs. *(note: <6 months time frame)*
- v) We have been promoting and using the guidelines for more than 6

months and have ongoing programs.

1. **If not using** (answers i or ii)**:** What are the reasons for not using the guidelines (Are there any barriers)?

________________________________________________________________________________________________________________________________________________________________________________________________________________________________________________________________________________________

1. **If using** (answers iii, iv or v)**:** How is your organization using the guidelines?

________________________________________________________________________________________________________________________________________________________________________________________________________________________________________________________________________________________

1. If using: Have you experienced any challenges or barriers to implementing the guidelines?

__________________________________________________________________________________________________________________________________________________________________________________________________________________

Thank you for answering our questions.

11. Would you like to receive additional information about this study for your records?

**** Yes **(Send Information Letter to organization)**

**** No

12. Would it be possible to contact you again in the future to discuss your organization’s adoption of the Alberta Nutrition Guidelines for children and youth?

**** Yes

**** No

Thank you again for participating in this survey.
